# Combined delivery of salinomycin and docetaxel by dual-targeting gelatinase nanoparticles effectively inhibits cervical cancer cells and cancer stem cells

**DOI:** 10.1080/10717544.2021.1886378

**Published:** 2021-03-04

**Authors:** Qin Wang, Ying-Tzu Yen, Chen Xie, Fangcen Liu, Qin Liu, Jia Wei, Lixia Yu, Lifeng Wang, Fanyan Meng, Rutian Li, Baorui Liu

**Affiliations:** a The Comprehensive Cancer Center of Drum Tower Hospital, Medical School of Nanjing University & Clinical Cancer Institute of Nanjing University, Nanjing, China; b Key Laboratory for Organic Electronics and Information Displays, Institute of Advanced Materials (IAM), Jiangsu National Synergetic Innovation Center for Advanced Materials (SICAM), Nanjing University of Posts & Telecommunications, Nanjing, China; c Department of pathology, Drum Tower Hospital, Medical School of Nanjing University, Nanjing, China

**Keywords:** Salinomycin, docetaxel, nanoparticles, cancer stem cells, cancer cells

## Abstract

Intra-tumor heterogeneity is widely accepted as one of the key factors, which hinders cancer patients from achieving full recovery. Especially, cancer stem cells (CSCs) may exhibit self-renewal capacity, which makes it harder for complete elimination of tumor. Therefore, simultaneously inhibiting CSCs and non-CSCs in tumors becomes a promising strategy to obtain sustainable anticancer efficacy. Salinomycin (Sal) was reported to be critical to inhibit CSCs. However, the poor bioavailability and catastrophic side effects brought about limitations to clinical practice. To solve this problem, we previously constructed gelatinase-stimuli nanoparticles composed of nontoxic, biocompatible polyethylene glycol-polycaprolactone (PEG-PCL) copolymer with a gelatinase-cleavable peptide Pro-Val-Gly-Leu-Iso-Gly (PVGLIG) inserted between the two blocks of the copolymer. By applying our “smart” gelatinase-responsive nanoparticles for Sal delivery, we have demonstrated specific accumulation in tumor, anti-CSCs ability and reduced toxicity of Sal-NPs in our previous study. In the present study, we synthesized Sal-Docetaxel-loaded gelatinase-stimuli nanoparticles (Sal-Doc NP) and confirmed single emulsion as the optimal method of producing Sal-Doc NPs (Sal-Doc SE-NP) in comparison with nanoprecipitation. Sal-Doc SE-NPs inhibited both CSCs and non-CSCs in mice transplanted with cervical cancer, and might be associated with enhanced restriction of epithelial-mesenchymal transition (EMT) pathway. Besides, the tumorigenic capacity and growing speed were obviously suppressed in Sal-Doc-SE-NPs-treated group in rechallenge experiment. Our results suggest that Sal-Doc-loaded gelatinase-stimuli nanoparticles could be a promising strategy to enhance antitumor efficacy and reduce side effects by simultaneously suppressing CSCs and non-CSCs.

## 1. Introduction

Despite numerous advances made in cancer treatment, chemotherapy remains of great importance to treat cervical cancer (CC) especially in developing countries, where the incidence of CC remains high (Venkatas & Singh, 2020). However, the nonspecific biodistribution and the following cellular damages impose considerable drawbacks in its clinical utility. Meanwhile, chemotherapy fails to manage disease progression and relapse in CC. There is a clear need for new strategies to treat CC.

Cervical Cancer stem cells (CCSCs), a part of cancer cells, are claimed to be responsible for tumor growth, disease relapse, and treatment resistance (Chandimali et al., 2020). Salinomycin (Sal) is a traditional therapeutic drug used to fight against bacteria and coccidian, which has drawn growing attention for its exceptional features in targeting and killing CSCs (Gupta et al., 2009; Zhang et al., 2011; Zhi et al., 2011; Zhou et al., 2013). Nevertheless, Sal is currently unsuitable for clinical usage due to its poor bioavailability and tremendous toxicity (Story & Doube, 2004; Boehmerle & Endres, 2011; Zhang et al., 2012; Dorne et al., 2013).

Chemotherapeutic failure is known to be associated with the promotion of cancer stemness (Milanovic et al., 2018). Docetaxel (Doc) is one of the first-line chemotherapeutic agents for recurrent or metastatic cervical cancer. However, it was reported to contribute to the enrichment of CSCs, overexpression of CSC-associated markers or pathways and decreased expression of epithelial-mesenchymal transition (EMT)-associated E-cadherin (Abubaker et al., 2013; Lu et al., 2015; Lv et al., 2019; Mohammad et al., 2019). Since tumors became more aggressive after resistant to Doc, there is an urgent need to develop a strategy for inhibiting CSCs and non-CSCs simultaneously (Zhang et al., 2020). Several studies have shown that Sal possesses favorable inhibitive capacity to Doc-resistant CSCs (Muntimadugu et al., 2016; Zhou et al., 2019). Besides, Sal could sensitize paclitaxel (PAC)-, docetaxel (DOC)-, vinblastine (VIN)-, or colchicine (COL)-treated cancer cell lines, suggesting that Sal has the potential to sensitize the cells treated with microtubule-targeting drugs (Kim et al., 2012). Hence, co-delivery of Sal and Doc could be a promising combination treatment for cervical cancer. Yet, the central issue of the combined strategy lies in their serious side effects.

With diversified drug-loading capacity as well as enhanced permeability and retention (EPR) effect, nanoparticles (NPs) can better encapsulate hydrophobic drugs and realize co-delivery. Since gelatinase is abundantly and specifically existed in most tumors, we have synthesized gelatinase-stimuli NPs by inserting the gelatinase-cleavable peptide Pro-Val-Gly-Leu-Iso-Gly (PVGLIG) into the bonding between polyethylene glycol (mPEG) and polycaprolactone (PCL) segments (mPEG-Pep-PCL) (Li et al., 2013). The copolymer that we used has been approved by FDA, which signifies the potential of the NPs for clinical application. When NPs were accumulated in tumors, the mPEG-peptide-PCL conjugates would be cleaved by gelatinase. The de-PEGylated NPs aggregate to form large particles and therefore able to be retained in tumor region. Also, aggregated particles enter tumor cells by endocytosis which may increase the cellular uptake and further improve the intracellular concentration of anticancer drugs. Hence, these ‘smart’ NPs possess passive tumor-targeting capacity through microenvironment stimuli strategies. In our previous study, we have proved that the NPs were capable of Sal delivery with high encapsulation efficacy and tumor targeting property. *In vivo* experiment demonstrated superior CCSCs inhibition effect by attenuating the EMT pathway and milder adverse events evidenced by no obvious pathological changes in the H and E staining of organs, body weight variations, and survival (Wang et al., 2014, 2020).

In this paper, based on our previous works, we constructed a co-delivery system loading with Sal and Doc by gelatinase-stimuli NPs. The combination of Sal and Doc not only sensitizes the antitumor effect of Doc, but also eliminates CCSCs and non-CCSCs in tumor tissues. The copolymer used to construct our NPs has been approved by FDA, indicating its translational value. The treatment of Sal-Doc NPs can minimize side effects and recurrence; meanwhile, it can maximize therapeutic efficacy (Scheme1). Therefore, our study highlights the potential of combined delivery of Sal and Doc with gelatinase-stimuli NPs to break through the limitation for transition to clinical application.

## 2. Materials and methods

### 2.1. Materials

mPEG-pep-PCL copolymer was prepared as reported previously (Li et al., 2013). Sal was purchased from China Institute of Veterinary Drug Control (Beijing, China). Human CD44 and CD133 antibodies were purchased from MiltenyiBiotec (Germany). Human VIM, E-cad, ZEB1, ZEB2 antibodies were purchased from Abcam (USA). LiVision plus Kit and DAB Kit were purchased from Fuzhou New Biotechnology Co., LTD (Fuzhou, China). Total Protein Extraction Kit, SDS-PAGE, Western Blotting Testing Kit, ECL Testin Kit and Braford Protein Testing Kit were purchased from Nanjing KeyGEN Biotech Co. (Nanjing, China). All the chemicals and testing kits were utilized followed by standard procedure and manufacturers’ instructions.

### 2.2. Salinomycin-docetaxel-loaded nanoparticles prepared by nanoprecipitation method and single emulsion method

#### 2.2.1. Sal-Doc NR-NP prepared by nanoprecipitation method with the best proportion of poloxamer 188 (F68)

According to our previous work (Wang et al., 2014), the optimal surfactant composition was determined as 5 mg of copolymer, 0.5 mg of Sal, and 2 mg of Doc. Dissolved in 200ul acetone, the mixture of copolymer, Sal, and Doc was added into 5 ml deionized water that contains 1% of F68, which was proved to be the optimal concentration for suspension of NPs. The Sal-Doc NR-NP was formed with the solution turned blue immediately after a quick stirring. The remaining acetone and non-incorporated drugs were removed by rotary vacuum evaporation and filtration respectively.

#### 2.2.2. Sal-Doc SE-NP prepared by single emulsion method

The Sal-Doc SE-NP was prepared by modified single emulsion method (Cui et al., 2014) with 10 mg copolymer, 1 mg Sal, and 4 mg Doc. Dissolved in 0.5 ml DCM, the mixture was emulsified by sonication with Microson XL2000 (Misonix, USA) in 1.5 ml 3% PVA solution (w/v) with 5 W of power for 60 seconds. The o/w emulsion was then emulsified with 2.5 ml of solution containing 0.5% (w/v) PVA with 2.5% of power by sonication for 10 seconds. The w/o/w emulsion was gently stirred at room temperature and filtered to remove dissociative drugs.

#### 2.2.3. Size and zeta potential analysis

Mean diameter and size distribution of the Sal-Doc SE-NP and Sal-Doc NR-NP were measured by dynamic light scattering (DLS) with Brookhaven BI-9000AT (Brookhaven Instruments Corporation, USA). Analysis of zeta potential was conducted by Zeta Plus (Brookhaven Instruments Corporation, USA), a laser Doppler anemometry. Each measurement was performed at least three times.

#### 2.2.4. Morphology

A drop of Sal-Doc SE-NP and Sal-Doc NR-NP suspension was dripped on a nitrocellulose-covered copper grid respectively and air-dried before observation under JEM-100S transmission electron microscope (TEM) (JEOL Ltd., Akishima-shi, Japan).

#### 2.2.5. Drug-loading capacity and encapsulation efficiency

To determine the drug-loading capacity of Sal-Doc SE-NP and Sal-Doc NR-NP, 1 ml of NPs were dried in the oven for about 12 hours, dissolved in 5 ml ethanol, and centrifuged (Table 1). The drug loading and encapsulation efficiency of Sal was determined by pre-High-Performance Liquid Chromatography. The supernatant was extracted and derivatized with 2,4-dinitrophenol (DNP) (Accelerating Scientific and Industrial Development Thereby Serving Humanity, China) in acidic medium at 55 °C for 30 minutes. The mixture was then analyzed with pre-High-Performance Liquid Chromatography (pre-HPLC) (Wang et al., 2020) and C18 column (Agilent Technologies, Ltd., USA) (mobile phase: methanol (HPLC grade, Merck):1.5%aqueous acetic acid (HPLC grade, Merck) = 93:7; flow rate: 1 mL/min; column temperature: 25 °C; injection volume: 20ul; detector wavelength: 392 nm; retention time: 10.75 min). The drug loading and encapsulation efficiency of DOC were analyzed by high-performance liquid chromatography (HPLC) system as we previously described (Liu et al., 2012). Chromatographic separation was achieved using a HC-C18 column (250, 4.6 mm, 5 mm, C18, Agilent Technologies, Palo Alto, USA) (mobile phase: double-distilled water (Millipore, Milford, USA): acetonitrile (HPLC grade, Merck) = 1:1; flow rate: 1.0 mL/min; column temperature: 35 °C; injection volume: 20ul; detector wavelength: 230 nm; retention time: 3.4 min).

**Table 1.** Parameters of HPLC for drug loading capacity analysis of Sal and Doc.

Drug	Mobile phase	Flow rate	Column temperature	Injection volume	Wavelength of detector
Sal	Methanol:1.5% aqueous acetic acid = 93:7	1 ml/min	25 °C	20 μl	392 nm
Doc	Acetonitrile: deionized water = 50:50 (v/v)	1 ml/min	30 °C	20 μl	230 nm

Sal: salinomycin; Doc: docetaxel.

The standard curves of Sal and Doc were measured by adopting standard drug solutions with concentration of 200, 120, 60, 40, 20, 50, 1, and 0.5 μg/ml respectively. All samples were filtered through 0.45-μm pore size filters (Millipore, Germany) before HPLC analysis. Drug-loading capacity and encapsulation efficiency were obtained by calculating the ratio between the drug in NPs and NPs or amount of drug offered (M_E_/M_T_×100%) (Wang et al., 2014).

#### 2.2.6. Stability evaluation

Sal-Doc SE-NP and Sal-Doc NR-NP were kept at room temperature. Sizes of particles were measured by DLS every 2 days for 15 days to assess the stability of NPs.

#### 2.2.7. *In vitro* drug release

*In vitro* release was investigated through dialysis method (Wang et al., 2020). In brief, 1 ml Sal-Doc SE-NP and Sal-Doc NR-NP were sealed in the dialysis bag with molecular weight cut off at 14000 Da. Immersed in 5 ml solution containing 0.01 M pH 7.4 PBS and 0.5% Tween 80 at 37 °C with constant shaking, the liquid was drawn out and replaced with the same volume of media at predetermined time points. The solution taken out was then examined by pre-HPLC and HPLC. All experiments were repeated for three times.

### 2.3. Cell lines and culture

Hela cells (Shanghai Institute of Biochemistry and Cell Biology, China), a highly metastatic human cervical cancer cell line (Arjomandnejad et al., 2014), were cultured by Rosewall Park Memorial Institute (RMPI) 1640 with 10% fetal bovine serum (FBS) at 37 °C in humidified chamber that contained 5% CO_2_ before implanted subcutaneously into the right posterior flanks of BALB/c mice.

### 2.4. *In vivo* antitumor effect of Sal-Doc SE-NP

4–5 weeks, 18–22 g male BALB/c mice (Animal Care Committee at Drum Tower Hospital, China) were housed under specific pathogen free (SPF) condition and acclimated to the laboratory environment a week prior to the start of the experiment. All animal studies were performed in compliance with guidelines of Animal Care Committee at Drum Tower Hospital.

Ten BALB/c mice were injected with 10^7^ Hela cells subcutaneously at the lower right axilla. The mice were sacrificed when the tumor volumes reached at 500mm^3^. Tumors were extracted and cut into 3 mm*3 mm*3 mm pieces. Another set of BALB/c mice were then embedded with the cut down pieces subcutaneously at the right axilla. The mice were randomly divided into seven groups with six mice each group (Table 2) when the tumor volumes reached about 100–200mm^3^ and the day was designated as ‘Day 0.’ Sizes of tumor were measured every other day and were calculated with the following formula:

$$\frac{{\text{maximum transverse witdh}}^{2}\times \text{maximum vertical width}}{2}$$

**Table 2.** Categorization of BALB/c mice on day 0.

Group	Treatment
Control	75% ethanol
Blank NPs	Blank NPs
2 + 8 NP	Sal-Doc SE-NP, Sal 2 mg/Kg and Doc 8 mg/Kg once
3 + 12 NP	Sal-Doc SE-NP, Sal 3 mg/Kg and Doc 12 mg/Kg once
4 + 16 NP	Sal-Doc SE-NP, Sal 4 mg/Kg and Doc 16 mg/Kg once
4 + 16 Free	Sal 1 mg/Kg and Doc 4 mg/Kg, every other day for 4 times
Doc Free	Doc 4 mg/Kg, every other day for 4 times

Sal = salinomycin; Doc = docetaxel; SE-NP = nanoparticles prepared by single emulsion method; NR-NP: nanoparticles prepared by nanoprecipitation method.

The survival of BALB/c mice after injection with free Sal-Doc (Sal 4 mg/kg + Doc 16 mg/kg) and Sal-Doc SE-NP (Sal 4 mg/kg + Doc 16 mg/kg) (5 mice for each group) were observed during the treatment.

The weights, appetite, mobility of BALB/c mice of each group were observed during the treatment. Organs were collected on day 14 for H and E staining to assess the systemic toxicity.

### 2.5. Immunohistochemistry (IHC) for CSCs markers and tumor proliferation ability

Paraffin-embedded tumor tissues of the mice from each group were cut into 4 mm thick. After deparaffinization and rehydration by xylene and ethanol respectively, the tissues were immersed in 3% hydrogen peroxide solution for 10 minutes to block endogenous peroxidases and boiled for 30 minutes in 10 mM citrate buffer solution (pH 6.0) for antigen retrieval. The tissues were incubated with 5% bovine serum albumin (BSA) for 45 minutes, followed by anti-PCNA, anti-Ki67, anti-Caspase3, anti-CD44, anti-CD133, anti-E-cadherin, and anti-VIM overnight at 4 °C. The specimens were visualized through Real Envision Detection Kit (GeneTech Shanghai Company Limited, China) after incubation with appropriate peroxidase-conjugated secondary antibody for 45 minutes at 37 °C. All slides were counter-stained with H and E.

### 2.6. Western blot (WB) assays for CSCs markers

The tumor tissues were grinded and suspended followed by washing with PBS solution twice. The cells were then lysed with RIPA Lysis Buffer (Beyotime Institute of Biotechnology, China) and protease inhibitor. Concentration of protein was determined by Pierce BCA Protein Assay Kit (Thermo Scientific, USA). Equivalent amounts of total protein (60ug) were boiled, electrophoretically separated with polyacrylamide gel at 80 volts and transferred to polyvinylidene difluoride membranes. Blocked with 5% emulsion prepared in PBS for 1 hour, the membranes were incubated overnight at 4 °C with 1:500 diluted primary antibodies (β-actin, CD44, CD133, E-cadherin, VIM, ZEB1 and ZEB2) and washed for 5 minutes thrice with 1:1000 diluted Tween 20-PBS. After incubation with appropriate 1:1000-diluted peroxidase-conjugated secondary antibodies for 45 minutes, membranes were washed with Tween-20-PBS for 10 minutes thrice. The membranes were then observed by Odyssey two-color infrared laser imaging system. The signal generated by b-actin was determined as internal control.

### 2.7. Flow cytometry for CSCs markers

Tumor cells obtained from mice of each group were analyzed under flow cytometry for CD44 expression. The tumors were resected and cut into pieces before digesting with collagenase. To obtain single cell suspension, the resulting pieces were then mixed with collagenase III followed by 15–20 minutes of incubation at 37 °C for dissociation. The samples were filtered through 40-mm cell strainer and washed with RPMI-1640 containing 20% FBS and PBS. The cells were then immune-stained with CD44-PE (BD Biosciences) at 4 °C for 20 minutes, washed and re-suspended in 500ul PBS before undergoing flow cytometry (BD FACS Aria II, BD Sciences, USA). The anti-IgG-PE antibody was used as control.

### 2.8. Tumor seeding study for tumor regeneration ability

The tumor tissues excised from murine models of each group were cut into 3*3*3 mm and placed in petri dish loaded with saline (Wu et al., 2018). The mice were randomly divided into six groups with six mice of each, anesthetized and embedded with prepared 3*3*3 mm tumor pieces subcutaneously at the right axilla. Formation rates and growth rates of the tumors were measured.

### 2.9. Statistical analysis

Statistical analysis was processed under two-tailed Student’s t-test. *p*-values less than .05 were considered statistically significant.

## 3. Results

### 3.1. Characteristics of Sal-Doc NP

#### 3.1.1. Sizes and morphology of Sal-Doc NP prepared by the two methods

**Figure 1.**
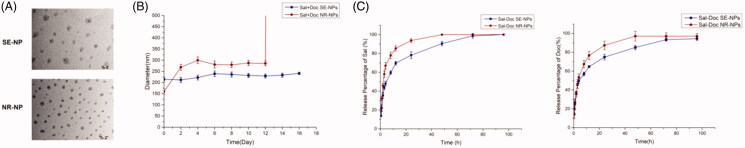
Characteristics of Sal-Doc NP. (A) Photos of Sal-Doc SE-NP and NR-NP under TEM; (B) Diameters of Sal-Doc SE-NP and NR-NP in 16 days; (C) Cumulative release of Sal and Doc respectively from Sal-Doc SE-NP and NR-NP. Sal = salinomycin; Doc = docetaxel; SE-NP: nanoparticles prepared by single emulsion method; NR-NP: nanoparticles prepared by nanoprecipitation method.

Size and polydispersity of Sal-Doc SE-NP and NR-NP measured by DLS were displayed in Table 3. Both Sal-Doc SE-NP and NR-NP were spherical surrounded with gray area under TEM as shown in Figure 1(A).

**Table 3.** Diameter and polydispersity of Sal-Doc SE-NP and NR-NP.

Nanoparticles	Diameter(nm)	Polydispersity
Sal-Doc SE-NP	214.5 ± 2.6	0.186 ± 0.013
Sal-Doc NR-NP	159.2 ± 3.2	0.063 ± 0.032

Sal: Salinomycin; Doc: Docetaxel; SE-NP: nanoparticles prepared by single emulsion method; NR-NP: nanoparticles prepared by nanoprecipitation method.

#### 3.1.2. Drug-loading content and encapsulation efficiency

Drug-loading capacity and encapsulation efficiency of Sal-Doc SE-NP and NR-NP were exhibited in Table 4. SE-NP exhibited higher drug entrapment efficiency than NR-NP.

**Table 4.** Drug-loading capacity and encapsulation efficiency of Sal-Doc SE- and NR-NP.

	Doc		Sal	
	Drug loading capacity (%)	Encapsulation efficiency (%)	Drug loading capacity (%)	Encapsulation efficiency (%)
Sal-Doc SE-NP	61.57 ± 3.9	10.26 ± 3.9	84.26 ± 7.3	7.66 ± 7.3
Sal-Doc NR-NP	35.32 ± 5.2	5.89 ± 5.2	65.17 ± 10.3	5.93 ± 10.3

Sal: salinomycin; Doc: docetaxel; SE-NP: nanoparticles prepared by single emulsion method; NR-NP: nanoparticles prepared by nanoprecipitation method.

#### 3.1.3. Stability evaluation

Diameters of nanoparticles measured within 16 days were displayed in Figure 1(B). Sizes of Sal-Doc SE-NP increased slightly while sizes of Sal-Doc NR-NP increased to 258 nm after 24 hours and enlarged notably on day 12.

#### 3.1.4. *In vitro* release of Sal-Doc NP

Pre-HPLC and HPLC were used to quantify released drugs. The cumulative release curve of both drugs was depicted in Figure 1(C). All NPs revealed a fast release at the initial stage followed by a sustained release of Sal and Doc. The drug release burst prominently in NR-NP with approximately 52.27% of Doc and 66.95% of Sal in the first 4 hours; 87.47% of Doc and 93.70% of Sal in the first 24 hours. On the other hand, the drug release of SE-NP exhibited a more sustainable pattern with approximately 54.98% of Doc and 48.00% of Sal in the first 4 hours; 74.91% of Doc and 88.00% of Sal in 24 hours.

#### 3.1.5. Toxicity evaluation of Sal-Doc SE-NP

The survival of mice after injection with free Sal-Doc (Sal 4 mg/kg + Doc 16 mg/kg) and Sal-Doc SE-NP (Sal 4 mg/kg + Doc 16 mg/kg) were shown in Table 5. Four mice died immediately after injection with free drugs while none of the mice in NPs group died at the end point of the experiment.

**Table 5.** The survival of mice after injection of different drug formulations (5 mice in each group).

	Day 0	Day 3	Day 6	Day 9
Free Doc + Sal	5	1	1	1
Sal-Doc SE-NP	5	5	5	5

Sal: salinomycin; Doc: docetaxel; SE-NP: nanoparticles prepared by single emulsion method.

### 3.2. *In vivo* anticancer efficacy of Sal-Doc SE-NP

**Figure 2.**
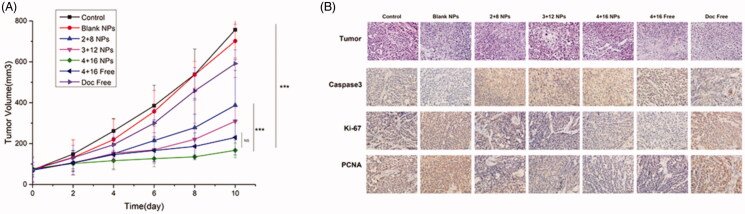
*In vivo* antitumor efficacy of Sal-Doc NP in a Hela tumor model. (A) Tumor inhibition rates of control, blank NPs, 2 + 8 NP, 3 + 12 NP, 4 + 16 NP, 4 + 16 Free and Doc Free 10 days after the first treatment; (B) H&E and IHC staining of control, blank NPs, 2 + 8 NP, 3 + 12 NP, 4 + 16 NP, 4 + 16 Free and Doc Free at the end point of the experiment.

Tumor inhibition rates of 2 + 8 NP, 3 + 12 NP, 4 + 16 NP, 4 + 16 Free and Doc Free 10 days after the first treatment were 48.8%, 59.6%, 77.9%, 69.6% and 21.85%, respectively (Figure 2(A)). The mice of 4 + 16 NP group showed the highest antitumor efficiency and the smallest tumor volumes (*p* <.001). No significant difference in tumor growing speed between 4 + 16 Free and 4 + 16 NP or 3 + 12 NP.

Pathological studies were conducted for further tumor analysis (Figure 2(B)). Dense tumor cells with large and hyperchromatic nuclei were observed in the control and blank NPs. Sporadic pink staining and karyopyknotic existed in Doc Free. The main composition in discrete necrotic regions of 4 + 16 Free and 3 + 12 NP was cell apoptosis, especially in 4 + 16 NP groups. Next, we studied cell growth with the proliferating cell nuclear antigen (PCNA), Ki-67, Caspase3 staining. Sal and Doc significantly increased the expression of Caspase3 and decreased the expression of Ki-67 and PCNA. Compared with Doc Free, 4 + 16 Free exhibited higher expression level of Caspase3 and lower expression of Ki-67 and PCNA.

### 3.3. *In vivo* toxicity

**Figure 3.**
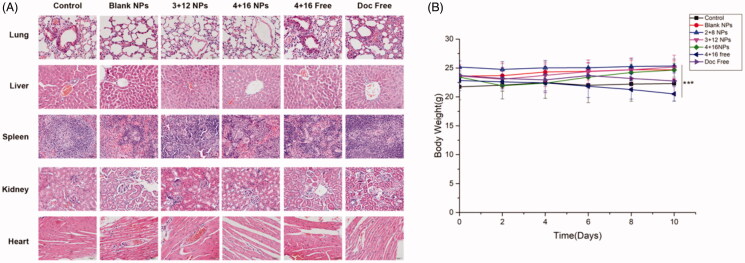
*In vivo* toxicity analysis of Sal-Doc SE-NP. (A) H&E staining of lugs, livers, spleens, kidneys, and hearts of control, blank NPs, 2 + 8 NP, 3 + 12 NP, 4 + 16 NP, 4 + 16 Free and Doc Free; (B) Mice weights of control, blank NPs, 2 + 8 NP, 3 + 12 NP, 4 + 16 NP, 4 + 16 Free and Doc Free.

H&E staining of specific organs were displayed in Figure 3(A) and no notable alterations in lung, liver, heart, kidney and spleen were observed in NPs treated groups. In 4 + 16 Free and Doc Free, obvious change in H and E staining of the lung was observed. Specifically, alveolar interstitial hyperemia, edema, and inflammatory cell infiltration were observed in lung tissues, which may lead to respiratory failure or even death. Compared with 4 + 16 Free and Doc Free, mice in 2 + 8 NP, 3 + 12 NP and 4 + 16 NP showed no significant difference in murine body weights (Figure 3(B)).

### 3.4. Sal-Doc SE-NP inhibits stem-like properties of hela cells *in vivo*

**Figure 4.**
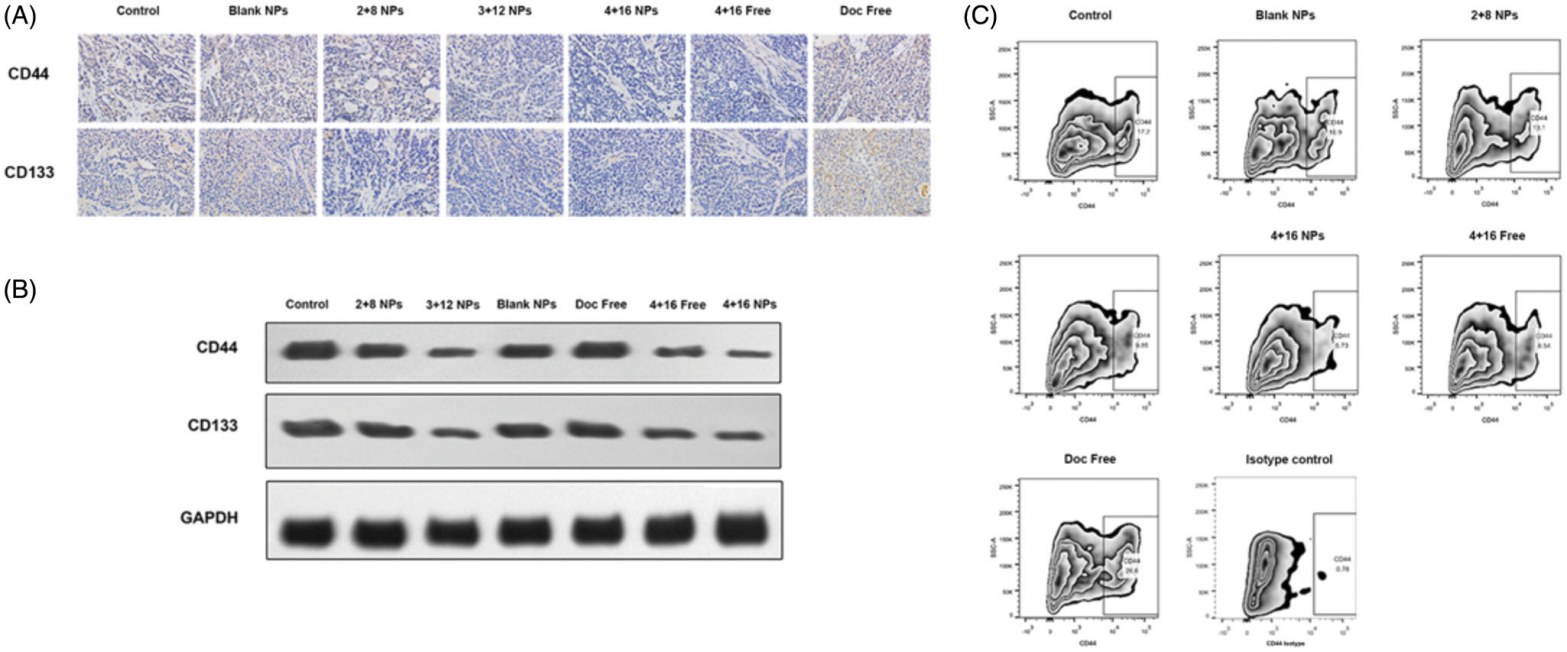
Sal-Doc SE-NP inhibits stem like properties of cervical cancer *in vivo*. (A) IHC staining of CD44 and CD133 of control, blank NPs, 2 + 8 NP, 3 + 12 NP, 4 + 16 NP, 4 + 16 Free and Doc Free; (B) Western blotting of CD44, CD133 and GAPDH; (C) Flow cytometry analysis for CD44 expression.

Mice received Sal-Doc SE-NP exhibited markedly lower levels of CD133 and CD44 than their control (Figure 4(A,B) and Supplemental Figure 1). Shown in Figure 4(C), Hela cells highly expressed CD44 (mean percentage 17.2%) in untreated group, and Doc Free treatment can increase the expression of CD44 (mean percentage 26.6%). On the other hand, 4 + 16 NP significantly decrease CD44 expression (mean percentage 5.73%), and 4 + 16 Free also lowered the percentage of CD44^+^ Hela cells to 8.54%.

### 3.5. Sal-Doc SE-NP inhibited EMT pathway of hela cells *in vivo*

**Figure 5.**
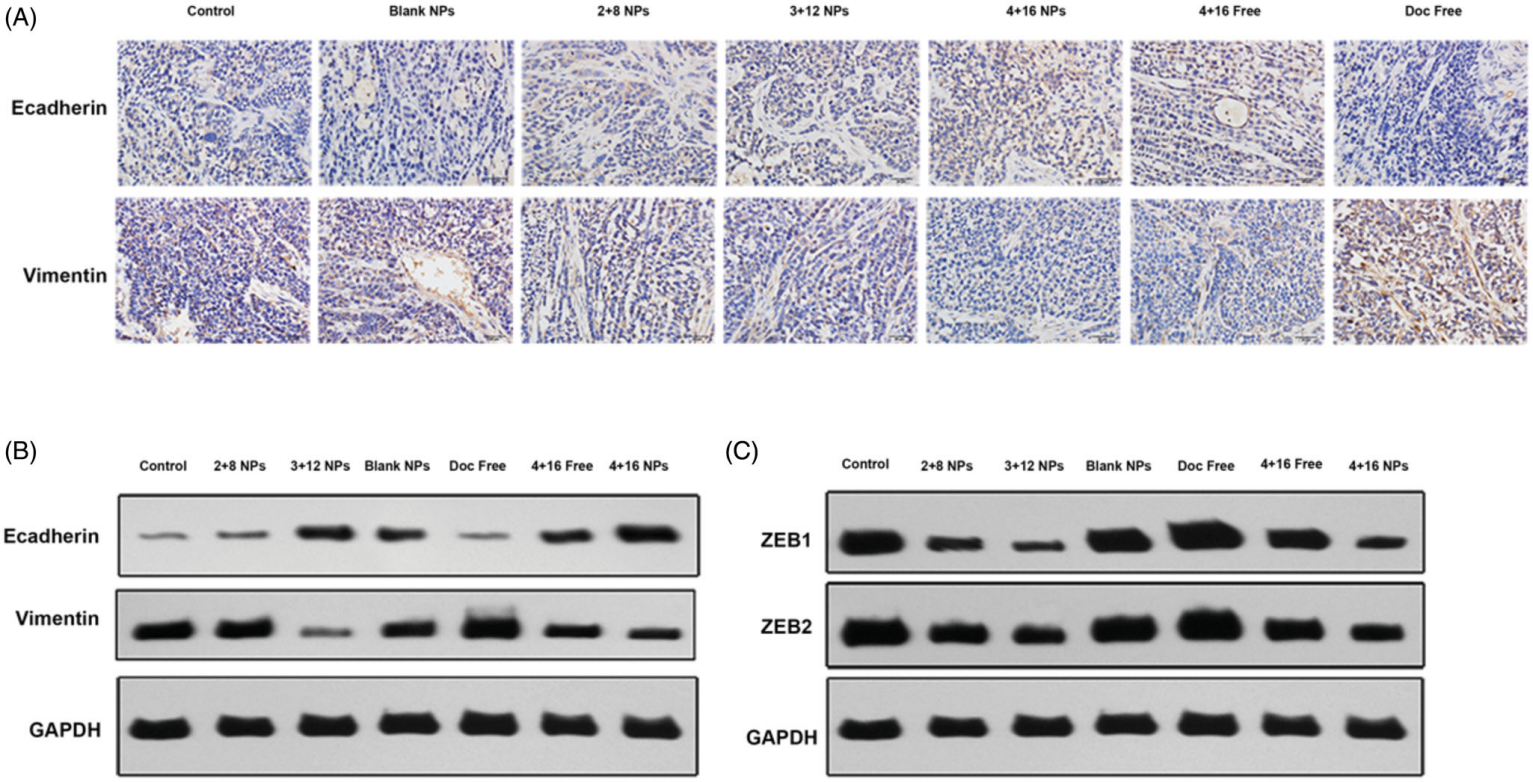
Mechanism of cancer stem cell inhibition by Sal-Doc SE-NP. (A) IHC staining for E-cad and Vimentin; (B) Western blotting of E-cad, Vimentin and GAPDH; (C) Western blotting of ZEB1, ZEB2and GAPDH.

IHC showed increased expression of E-cadherin and reduced expression of VIM (Figure 5(A,B) and Supplemental Figure 1). WB analysis revealed that the expression of ZEB1 and ZEB2 increased after Doc Free, while Sal-Doc SE-NP could decrease the ZEB1 and ZEB2 expression (Figure 5(C) and Supplemental Figure 1).

### 3.6. Sal-Doc SE-NP suppress the tumor regeneration ability of hela cells

**Figure 6.**
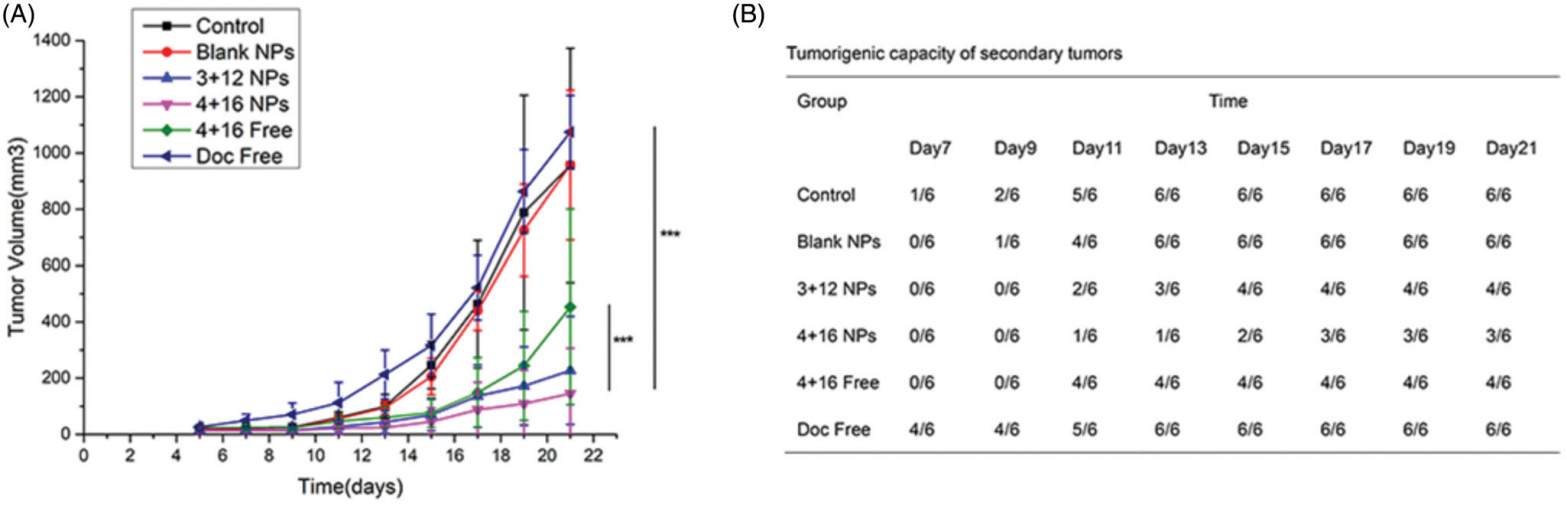
Tumor rechallenge assay and growth curves of secondary tumors. (A) Tumor volumes; (B) Tumorigenic capacity of secondary tumors.

To further investigate the biological activity of Sal-Doc SE-NP on CCSCs and EMT characteristics of tumor cells from CC tissues after anticancer treatment, we transplanted fresh CC specimens of 6 groups on the nude mice. Then we studied tumorigenic capacity and growth speed of these tumor-bearing mice under the same conditions. Figure 6(B) shows that tumor spheres could be observed on 100% (6/6) mice in Control group on the 13th days after transplantation, while 50% (3/6) mice in 3 + 12 NP group, 16.6% (1/6) mice in 4 + 16 NP and 66.6% (4/6) mice in 4 + 16 Free exhibited tumor formation. Before the endpoint of the experiment, three mice in 3 + 12 NP group did not develop tumors. Besides, 66.6% (4/6) mice in Doc regenerated on 7^th^ days after transplantation and 100% (6/6) mice on the 13^th^ days after transplantation and the growing speed significantly increased as shown in Figure 6(A). 4 + 16 NP group showed delay growth in comparison with the NS group (*p* < .001).

## 4. Discussion

Nanoparticles have been widely investigated in the treatment of cancer due to their special characteristics (Wang et al., 2017). Therefore, various nanoparticles were designed to be encapsulated with anticancer drugs (Wang et al., 2018). However, most of the studies put more focus on synthesizing novel materials as carriers than the selection of antitumor drugs they delivered. Applying appropriate nanoparticles for drug delivery in cancer therapy can not only increase chemotherapeutic effects, but also decrease drug-induced side effects. Thus, choosing the right chemotherapy regime to be encapsulated in nanoparticles based on anti-cancer mechanism is of great importance. Doc is one of the key agents in standard treatment of cervical cancer (Eswaran et al., 2018). Though cancer cells are sensitive to Doc, CSCs are not, which leads to tumor recurrence after Doc treatment. Combinatorial chemotherapy becomes increasingly needed, and the drug adopted for the combination is required to work through different mechanisms in order to effectively function in synergy (Wang, 2020). Thus, the usage of co-delivery systems renders greater therapeutic effect compared to a single treatment modality (Chen et al., 2019; Lin et al., 2019). Besides, the combined delivery system can incorporate both hydrophilic and hydrophobic drugs and achieve higher concertation of these drugs in an appropriate ratio (Cheng et al., 2017). Sal is an effective CSCs inhibitor (Lee et al., 2017) and may enhance the apoptotic capability of Doc (Wang et al., 2016). Co-delivery of Sal and Doc suppresses both cancer cells and CSCs and may be a promising approach in overcoming cancer recurrence induced by resistant cell population. The major obstacle for combination therapy lies in lack of specificity that may cause catastrophic side effects (Li et al., 2020). To solve the problem, nanoparticles were developed to deliver multiple drugs (Hu & Zhang, 2012; Glasgow & Chougule, 2015). In our previous study, we successfully encapsulated Sal into our MMP-stimuli nanoparticles and demonstrated reduced side effect of Sal. To comprehensively study the influence of Sal-Doc NP, we synthesized the Sal-Doc NP with single emulsion method and nanoprecipitation method respectively and confirmed the optimal method for Sal-Doc NP fabrication. Identification of satisfactory side effects inhibition as well as antitumor potency were also proved by *in vivo* evaluation.

The 4 + 16 NP showed the highest antitumor efficiency with the smallest tumor volumes, which demonstrated the sustainable release and targeted delivery capacity of gelatinase-responsive NPs. Besides, there were no difference between 4 + 16 Free and 3 + 12 NP, which further demonstrated the remarkable antitumor effects brought by 4 + 16 NP. Furthermore, the discrete necrotic regions were particularly obvious with cell apoptosis in 4 + 16 NP, which explained the smallest tumor volumes from pathology. Treating with Sal and Doc could induce tumor apoptosis and inhibit tumor proliferation, evidenced by significant increase in the expression of Caspase3 and decrease in the expression of Ki-67 and PCNA. As shown in Figure 2(B), 4 + 16 Free showed superior tumor inhibition while 4 + 16 NP showed the highest expression in Caspase3 and lowest expression of Ki-67 and PCNA compared with Doc Free, which confirmed the target capacity and sustainable release of NPs.

EMT, characterized by the loss of cell-cell adhesion as well as the initiation of invasion and metastasis, plays a critical role in resistance to most conventional therapeutics (Gaponova et al., 2020; Garcia-Mayea et al., 2020). The hallmarks of EMT are loss of E-cadherin expression, gain of mesenchymal markers, and increased mortality. In this study, IHC and WB were conducted to particularly analyze the role of Sal-Doc SE-NP played in EMT pathway. From Figure 5(A and B), increased expression of E-cadherin and reduced expression of VIM indicated the inhibition property of Sal on EMT process. A surge of papers reported that EMT was regulated by several transcription factors, including ZEB1and ZEB2, which may inhibit the expression of epithelial phenotype and repress E-cadherin transcription (Zheng et al., 2015; Zhang et al., 2018, 2019). Negative modulation of ZEB1 and ZEB2 expression was also observed through WB. Thus, Sal-Doc SE-NP may reverse EMT pathway by E-cadherin overexpression and enhance the antitumor efficacy of Doc.

A series of studies have reported that EMT is associated with the emergence of CSCs, which also recognized as the basis of tumor heterogeneity. Epithelial cells under EMT process often overexpress CSCs phenotype and exhibit CSCs characteristics (Wang et al., 2020; Almotiri et al., 2020). In this study, the expression of CCSC-related markers CD44 and CD133 were examined for evaluating the impact on stem-like properties by IHC, WB, and flow cytometry (Takaishi et al., 2009; Ortiz-Sánchez et al., 2016; Li et al., 2017; Organista-Nava et al., 2019). Hela cells highly expressed CD44 and the expression could be potentiated by Doc. Co-delivery of Sal and Doc decreased CD44 expressions. Moreover, Sal-Doc SE-NP exhibited markedly lower level of CD133 and CD44 especially in 4 + 16 NP, suggesting that 4 + 16 NP exerted the strongest inhibition efficacy of CCSCs. Together, CD44 down-regulation and E-cadherin overexpression indicate Sal-Doc SE-NP could inhibit CSCs or induce CSCs transforming, which is one key target for suppression of cervical tumor progression.

The tumorigenic capacity and growing speed of these tumor-bearing mice under the same condition were also investigated. From Figure 6(A,B), all the mice in single Doc treatment group developed tumors 13 days after transplantation and the growing speed significantly increased, which demonstrated that single Doc treatment may enrich CCSCs and cause tumor recurrence. However, combination therapy may decrease tumorigenic capacity and growing speed. 4 + 16 NP group showed the most profound inhibitory effect with only one mouse observed for tumor formation 13 days after transplantation of secondary CC cells and the slowest tumor growing pattern. Besides, 3 + 12 NP group showed a lower percentage of tumor formation than 4 + 16 Free group, which highlighted the sustainable release and enhanced accumulation of Sal-Doc SE-NP. All of these supported the predominant synergetic inhibitory effect of Sal and Doc on CCSCs and cancer cells.

In this study, the gelatinases-stimuli property of nanoparticles accomplishes simultaneous delivery of Sal and Doc with better targeted efficiency. Zetasizer Nano ZS Analyzer and TEM suggesting that Sal and Doc have been properly encapsulated. A general consensus existed that larger sizes of particles could spare a larger space for encapsulation (Ospina-Villa et al., 2019; Shahriari et al., 2019). It is consistent with our results that SE-NP displayed larger sizes and higher encapsulation efficiency in comparison with NR-NP. On the other hand, the lower encapsulation efficiency of NR-NP may be contributed to the residue surfactant molecules failed to be washed away on the surface of the particles (Dong & Feng, 2004). The particle size of Sal-Doc NR-NP increased notably after the first 24 hours in 16-day measurement, suggesting that NR-NP had a tendency to aggregate and remained unstable. SE-NP, on the contrary, increased slightly in size, which indicates a better stability. The release curve of Sal and Doc loaded on NR-NP presented a burst release. The result may be ascribed to the smaller particle size and surfactant molecules on the particle surface. In addition, the release pattern of Sal and Doc from both NPs was biphasic. In other words, the release profile could be roughly divided into two phases: the burst phase completed within the first 12 hours and the sustained phase for up to 96 hours after the initial burst.

Side effects are the major obstacle in the application of drug combinations. In the toxicity evaluation study, surprisingly, we discovered that the survival time significantly improved with no mice died in Sal-Doc SE-NP group at the end point of the experiment while four mice died immediately after injection of free Sal and Doc (Table 3). Therefore, it was verified that encapsulating Sal and Doc into SE-NP can reduce their side effects. The possible mechanisms may be: (a) Sal-Doc SE-NP could increase therapeutic concentration through targeted delivery to tumor tissues; (b) the retention of release kept normal tissues stay below lethal threshold; (c) the abrogation of ethanol as solvent to dissolve Doc prevented additional side effects. Next, from *in vivo* toxicity study, mice in 2 + 8 NP, 3 + 12 NP and 4 + 16 NP displayed no difference of note comparing with 4 + 16 Free and Doc Free in body weights and staining of organs. This highlighted the *in vivo* safety of 2 + 8 NP, 3 + 12 NP and 4 + 16 NP.

Altogether, the drug delivery system presented in this study is proposed to include the following advantages: (1) simultaneously delivering Sal and Doc into the same sites of tumor with sustainable release based on EPR effect and gelatinases-stimuli strategy following systemic injection; (2) the gelatinases-targeting strategy to deliver Sal to CSCs cells; (3) synergistically inhibiting tumor growth through killing non-CSC cancer cells by Doc, killing CSCs, reversing EMT, enhancing Doc sensitivity, and avoiding tumor recurrence as well as clinical relapse by Sal.

## 5. Conclusion

In summary, we constructed a delivery system consisting of Sal, Doc, and gelatinase-stimuli NPs and confirmed its efficacy *in vivo* with inhibition of tumors, little changes in pathology, and decreased expressions of CCSC markers. The possible mechanism of the particles could be suppression of EMT pathway. Also, we firstly constructed Sal-Doc SE-NP and demonstrated their ability to reduce side effects and improve chemotherapeutic efficacy. Last but not least, it provides a proper model for the application of nanoparticles in tumor treatment.

## Supplementary Material

Supplemental Material

## References

[CIT0001] Almotiri A, Alzahrani HAA, Menendez-Gonzalez JB, et al. (2020). Zeb1 modulates hematopoietic stem cell fates required for suppressing acute myeloid leukemia. J Clin Invest 131(1):e129115. 10.1172/JCI129115 PMC7773410 33108352

[CIT0002] Abubaker K, Latifi A, Luwor R, et al. (2013). Short-term single treatment of chemotherapy results in the enrichment of ovarian cancer stem cell-like cells leading to an increased tumor burden. Mol Cancer 12:24. 23537295 10.1186/1476-4598-12-24 PMC3668985

[CIT0003] Arjomandnejad M, Muhammadnejad A, Haddadi M, et al. (2014). HeLa cell line xenograft tumor as a suitable cervical cancer model: growth kinetic characterization and immunohistochemistry array. Arch Iran Med 17:273–277. 24724604

[CIT0004] Boehmerle W, Endres M. (2011). Salinomycin induces calpain and cytochrome c-mediated neuronal cell death. Cell Death Dis 2:e168. 21633391 10.1038/cddis.2011.46 PMC3168989

[CIT0005] Chandimali N, Koh H, Kim J, et al. (2020). BRM270 targets cancer stem cells and augments chemo-sensitivity in cancer. Oncol Lett 20:103. 32831922 10.3892/ol.2020.11964 PMC7439126

[CIT0006] Chen H, Zeng X, Tham HP, et al. (2019). NIR-light-activated combination therapy with a precise ratio of photosensitizer and prodrug using a host-guest strategy. Angew Chem Int Ed Engl 58:7641–7646. 30980463 10.1002/anie.201900886

[CIT0007] Cheng L, Jiang D, Kamkaew A, et al. (2017). Renal-clearable PEGylated porphyrin nanoparticles for image-guided photodynamic cancer therapy. Adv Funct Mater 27:1702928. 29151826 10.1002/adfm.201702928 PMC5687274

[CIT0008] Cui FB, Li RT, Liu Q, et al. (2014). Enhancement of radiotherapy efficacy by docetaxel-loaded gelatinase-stimuli PEG-Pep-PCL nanoparticles in gastric cancer. Cancer Lett 346:53–62. 24333735 10.1016/j.canlet.2013.12.002

[CIT0009] Dong Y, Feng SS. (2004). Methoxy poly(ethylene glycol)-poly(lactide) (MPEG-PLA) nanoparticles for controlled delivery of anticancer drugs. Biomaterials 25:2843–2849. 14962562 10.1016/j.biomaterials.2003.09.055

[CIT0010] Dorne JLCM, Fernández-Cruz ML, Bertelsen U, et al. (2013). Risk assessment of coccidostatics during feed cross-contamination: animal and human health aspects. Toxicol Appl Pharmacol 270:196–208. 21215766 10.1016/j.taap.2010.12.014

[CIT0011] Eswaran P, Kalyan S, Crystal J. (2018). Concurrent chemoradiation with nano- paclitaxel and carboplatin in locally advanced cervical cancer: A study at quaternary care medical center. Ann Oncol 29:ix81.

[CIT0012] Gaponova AV, Rodin S, Mazina AA, Volchkov PV. (2020). Epithelial-mesenchymal transition: role in cancer progression and the perspectives of antitumor treatment. Acta Naturae 12:4–23. 10.32607/actanaturae.11010 PMC7604894 33173593

[CIT0013] Garcia-Mayea Y, Mir C, Carballo L, et al. (2020). TSPAN1: A novel protein involved in head and neck squamous cell carcinoma chemoresistance. Cancers 12:3269. 10.3390/cancers12113269 PMC7694336 33167355

[CIT0014] Glasgow MD, Chougule MB. (2015). Recent developments in active tumor targeted multifunctional nanoparticles for combination chemotherapy in cancer treatment and imaging. J Biomed Nanotechnol 11:1859–1898. 26554150 10.1166/jbn.2015.2145 PMC4816444

[CIT0015] Gupta PB, Onder TT, Jiang G, et al. (2009). Identification of selective inhibitors of cancer stem cells by high-throughput screening. Cell 138:645–59. 19682730 10.1016/j.cell.2009.06.034 PMC4892125

[CIT0016] Hu CM, Zhang L. (2012). Nanoparticle-based combination therapy toward overcoming drug resistance in cancer. Biochem Pharmacol 83:1104–1111. 22285912 10.1016/j.bcp.2012.01.008

[CIT0017] Kim JH, Yoo HI, Kang HS, et al. (2012). Salinomycin sensitizes antimitotic drugs-treated cancer cells by increasing apoptosis via the prevention of G2 arrest. Biochem Biophys Res Commun 418:98–103. 22244892 10.1016/j.bbrc.2011.12.141

[CIT0018] Lee HG, Shin SJ, Chung HW, et al. (2017). Salinomycin reduces stemness and induces apoptosis on human ovarian cancer stem cell. J Gynecol Oncol 28:e14. 27894167 10.3802/jgo.2017.28.e14 PMC5323284

[CIT0019] Li K, Zhan W, Jia M, et al. (2020). Dual loading of nanoparticles with doxorubicin and icotinib for the synergistic suppression of non-small cell lung cancer. Int J Med Sci 17:390–402. 32132874 10.7150/ijms.39172 PMC7053357

[CIT0020] Li L, Cui D, Ye L, et al. (2017). Codelivery of salinomycin and docetaxel using poly(D,L-lactic-co-glycolic acid)-poly(ethylene glycol) nanoparticles to target both gastric cancer cells and cancer stem cells. Anticancer Drugs 28:989–1001. 28692437 10.1097/CAD.0000000000000541

[CIT0021] Li R, Wu W, Liu Q, et al. (2013). Intelligently targeted drug delivery and enhanced antitumor effect by gelatinase-responsive nanoparticles. PLoS One 8:e69643. 23936062 10.1371/journal.pone.0069643 PMC3728361

[CIT0022] Lin YX, Wang Y, An HW, et al. (2019). Peptide-based autophagic gene and cisplatin co-delivery systems enable improved chemotherapy resistance. Nano Lett 19:2968–2978. 30924343 10.1021/acs.nanolett.9b00083

[CIT0023] Liu Q, Li RT, Qian HQ, et al. (2012). Gelatinase-stimuli strategy enhances the tumor delivery and therapeutic efficacy of docetaxel-loaded poly(ethylene glycol)-poly(varepsilon-caprolactone) nanoparticles. Int J Nanomedicine 7:281–295. 22287839 10.2147/IJN.S26697 PMC3265997

[CIT0024] Lu H, Samanta D, Xiang L, et al. (2015). Chemotherapy triggers HIF-1-dependent glutathione synthesis and copper chelation that induces the breast cancer stem cell phenotype. Proc Natl Acad Sci USA 112:E4600–4609. 26229077 10.1073/pnas.1513433112 PMC4547233

[CIT0025] Lv Y, Cang W, Li Q, et al. (2019). Erlotinib overcomes paclitaxel-resistant cancer stem cells by blocking the EGFR-CREB/GRβ-IL-6 axis in MUC1-positive cervical cancer. Oncogenesis 8:70. 31772161 10.1038/s41389-019-0179-2 PMC6879758

[CIT0026] Milanovic M, Fan DNY, Belenki D, et al. (2018). Senescence-associated reprogramming promotes cancer stemness. Nature 553:96–100. 29258294 10.1038/nature25167

[CIT0027] Mohammad IS, Teng C, Chaurasiya B, et al. (2019). Drug-delivering-drug approach-based codelivery of paclitaxel and disulfiram for treating multidrug-resistant cancer. Int J Pharm 557:304–13. 30599232 10.1016/j.ijpharm.2018.12.067

[CIT0028] Muntimadugu E, Kumar R, Saladi S, et al. (2016). CD44 targeted chemotherapy for co-eradication of breast cancer stem cells and cancer cells using polymeric nanoparticles of salinomycin and paclitaxel. Colloids Surf B Biointerfaces 143:532–46. 27045981 10.1016/j.colsurfb.2016.03.075

[CIT0029] Organista-Nava J, Gómez-Gómez Y, Garibay-Cerdenares OL, et al. (2019). Cervical cancer stem cell-associated genes: Prognostic implications in cervical cancer. Oncol Lett 18:7–14. 31289465 10.3892/ol.2019.10307 PMC6540231

[CIT0030] Ortiz-Sánchez E, Santiago-López L, Cruz-Domínguez VB, et al. (2016). Characterization of cervical cancer stem cell-like cells: phenotyping, stemness, and human papilloma virus co-receptor expression. Oncotarget 7:31943–31954. 27008711 10.18632/oncotarget.8218 PMC5077987

[CIT0031] Ospina-Villa JD, Gómez-Hoyos C, Zuluaga-Gallego R, Triana-Chávez O. (2019). Encapsulation of proteins from Leishmania panamensis into PLGA particles by a single emulsion-solvent evaporation method. J Microbiol Methods 162:1–7. 31078626 10.1016/j.mimet.2019.05.004

[CIT0032] Shahriari M, Taghdisi SM, Abnous K, et al. (2019). Synthesis of hyaluronic acid-based polymersomes for doxorubicin delivery to metastatic breast cancer. Int J Pharm 572:118835–118835. 31726198 10.1016/j.ijpharm.2019.118835

[CIT0033] Story P, Doube A. (2004). A case of human poisoning by salinomycin, an agricultural antibiotic. N Z Med J 117:U799. 15107902

[CIT0034] Takaishi S, Okumura T, Tu S, et al. (2009). Identification of gastric cancer stem cells using the cell surface marker CD44. Stem Cells 27:1006–1020. 19415765 10.1002/stem.30 PMC2746367

[CIT0035] Venkatas J, Singh M. (2020). Cervical cancer: a meta-analysis, therapy and future of nanomedicine. Ecancer Med Sci 14:1111. 10.3332/ecancer.2020.1111 PMC7581334 33144879

[CIT0036] Wang G, Wang Z, Li C, et al. (2018). RGD peptide-modified, paclitaxel prodrug-based, dual-drugs loaded, and redox-sensitive lipid-polymer nanoparticles for the enhanced lung cancer therapy. Biomed Pharmacother 106:275–284. 29966971 10.1016/j.biopha.2018.06.137

[CIT0037] Wang J. (2020). Combination treatment of cervical cancer using folate-decorated, ph-sensitive, carboplatin and paclitaxel co-loaded lipid-polymer hybrid nanoparticles. Drug Des Devel Ther 14:823–832. 10.2147/DDDT.S235098 PMC7049774 32161442

[CIT0038] Wang Q, Liu F, Wang L, et al. (2020). Enhanced and prolonged antitumor effect of salinomycin-loaded gelatinase-responsive nanoparticles via targeted drug delivery and inhibition of cervical cancer stem cells. Int J Nanomedicine 15:1283–1295. 32161458 10.2147/IJN.S234679 PMC7049776

[CIT0039] Wang Q, Wu P, Ren W, et al. (2014). Comparative studies of salinomycin-loaded nanoparticles prepared by nanoprecipitation and single emulsion method. Nanoscale Res Lett 9:351. 25147486 10.1186/1556-276X-9-351 PMC4134115

[CIT0040] Wang S, Meng X, Dong Y. (2017). Ursolic acid nanoparticles inhibit cervical cancer growth in vitro and in vivo via apoptosis induction. Int J Oncol 50:1330–1340. 28259944 10.3892/ijo.2017.3890

[CIT0041] Wang T, Narayanaswamy R, Ren H, Torchilin VP. (2016). Combination therapy targeting both cancer stem-like cells and bulk tumor cells for improved efficacy of breast cancer treatment. Cancer Biol Ther 17:698–707. 27259361 10.1080/15384047.2016.1190488 PMC4990396

[CIT0042] Wang X, Chang Y, Gao M, Zhang F. (2020). Wogonoside attenuates cutaneous squamous cell carcinoma by reducing epithelial-mesenchymal transition/invasion and cancer stem-like cell property. Onco Targets Ther 13:10097–10109. 33116592 10.2147/OTT.S251806 PMC7553668

[CIT0043] Wang Z, Sun M, Li W, et al. (2020). A novel CD133- and EpCAM-targeted liposome with redox-responsive properties capable of synergistically eliminating liver cancer stem cells. Front Chem 8:649. 32850663 10.3389/fchem.2020.00649 PMC7431664

[CIT0044] Wu P, Liu Q, Wang Q, et al. (2018). Novel silk fibroin nanoparticles incorporated silk fibroin hydrogel for inhibition of cancer stem cells and tumor growth. Int J Nanomedicine 13:5405–5418. 30271137 10.2147/IJN.S166104 PMC6149978

[CIT0045] Zhang G-N, Liang Y, Zhou L-J, et al. (2011). Combination of salinomycin and gemcitabine eliminates pancreatic cancer cells. Cancer Lett 313:137–44. 22030254 10.1016/j.canlet.2011.05.030

[CIT0046] Zhang H, Zhang Y, Chen C, et al. (2018). A double-negative feedback loop between DEAD-box protein DDX21 and Snail regulates epithelial-mesenchymal transition and metastasis in breast cancer. Cancer Lett 437:67–78. 30165191 10.1016/j.canlet.2018.08.021

[CIT0047] Zhang S, Cheng J, Quan C, et al. (2020). circCELSR1 (hsa_circ_0063809) contributes to paclitaxel resistance of ovarian cancer cells by regulating FOXR2 expression via miR-1252. Mol Ther Nucleic Acids 19:718–30. 31945729 10.1016/j.omtn.2019.12.005 PMC6965731

[CIT0048] Zhang Y, Zhang H, Wang X, et al. (2012). The eradication of breast cancer and cancer stem cells using octreotide modified paclitaxel active targeting micelles and salinomycin passive targeting micelles. Biomaterials 33:679–91. 22019123 10.1016/j.biomaterials.2011.09.072

[CIT0049] Zhang Z, Xu J, Liu B, et al. (2019). Ponicidin inhibits pro-inflammatory cytokine TNF-α-induced epithelial-mesenchymal transition and metastasis of colorectal cancer cells via suppressing the AKT/GSK-3β/Snail pathway. Inflammopharmacology 27:627–638. 30244296 10.1007/s10787-018-0534-5

[CIT0050] Zheng M, Jiang Y-p, Chen W, et al. (2015). Snail and Slug collaborate on EMT and tumor metastasis through miR-101-mediated EZH2 axis in oral tongue squamous cell carcinoma. Oncotarget 6:6797–6810. 25762643 10.18632/oncotarget.3180 PMC4466650

[CIT0051] Zhi QM, Chen XH, Ji J, et al. (2011). Salinomycin can effectively kill ALDH(high) stem-like cells on gastric cancer. Biomed Pharmacother 65:509–15. 21996439 10.1016/j.biopha.2011.06.006

[CIT0052] Zhou J, Li P, Xue X, et al. (2013). Salinomycin induces apoptosis in cisplatin-resistant colorectal cancer cells by accumulation of reactive oxygen species. Toxicol Lett 222:139–45. 23916687 10.1016/j.toxlet.2013.07.022

[CIT0053] Zhou J, Sun M, Jin S, et al. (2019). Combined using of paclitaxel and salinomycin active targeting nanostructured lipid carriers against non-small cell lung cancer and cancer stem cells. Drug Deliv 26:281–9. 30880491 10.1080/10717544.2019.1580799 PMC6427498

[CIT0054] Zhou S, Wang F, Wong ET, et al. (2013). Salinomycin: a novel anti-cancer agent with known anti-coccidial activities. Curr Med Chem 20:4095–101. 23931281 10.2174/15672050113109990199 PMC4102832

