# Supplementary figures and images for: Combined delivery of salinomycin and docetaxel by dual-targeting gelatinase nanoparticles effectively inhibits cervical cancer cells and cancer stem cells
Source: Drug Deliv. 2021 Mar 4;28(1):510–9. doi: 10.1080/10717544.2021.1886378 (PMC7935125; doi:10.1080/10717544.2021.1886378)

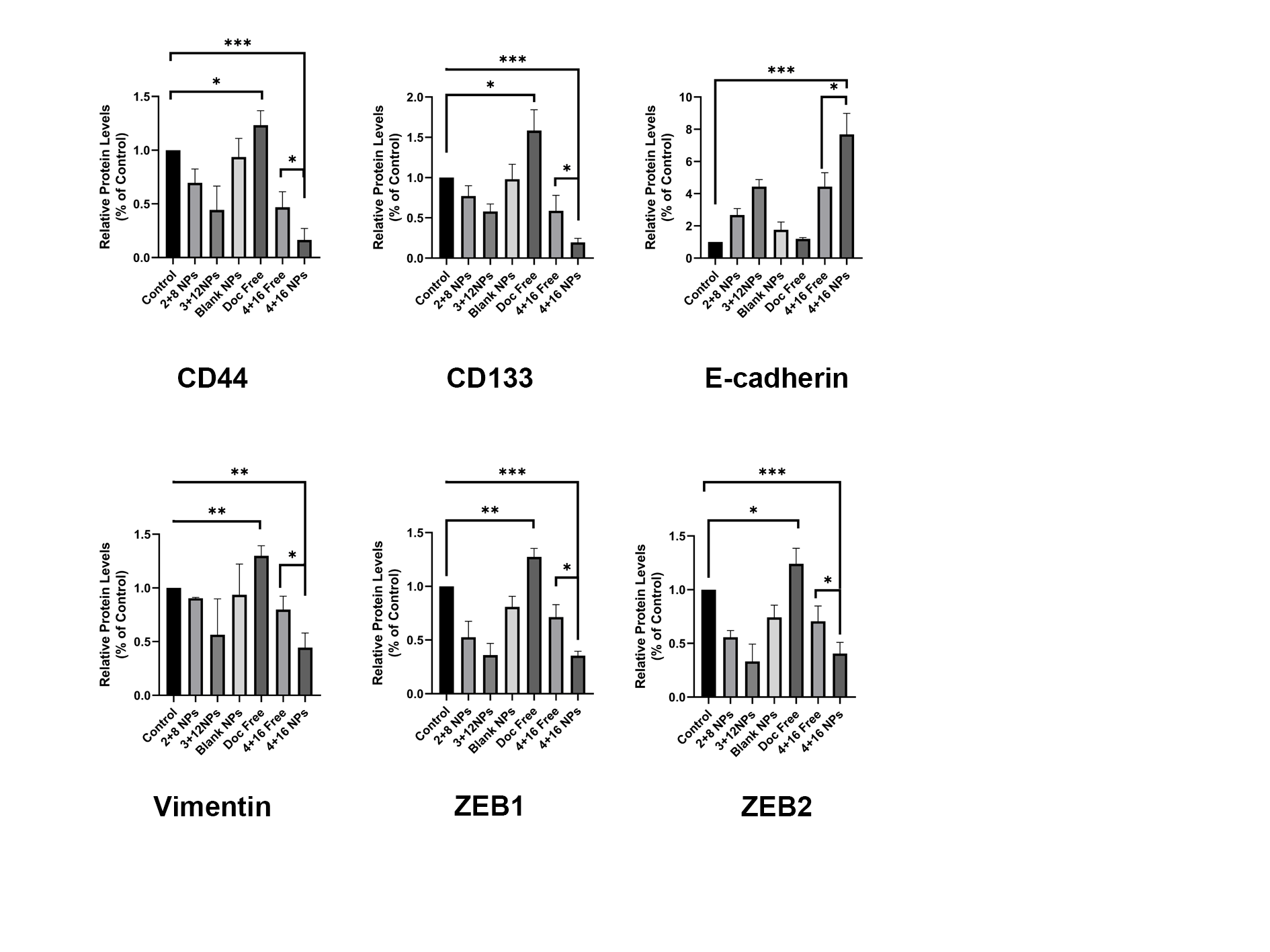

Supplement: Supplemental Material [file IDRD_A_1886378_SM1312.tif]
